# Survival beyond treatment: patterns and determinants of disease-free survival in cervical cancer at a tertiary care center in India

**DOI:** 10.3389/fonc.2026.1872738

**Published:** 2026-07-10

**Authors:** Bajarang Bahadur, Sunil Choudhary, Deshna Oswal, Tej Singh, Shraddha Chaurasiya, Mayank Singh

**Affiliations:** 1 Department of Community Medicine, Employees' State Insurance Corporation Medical College and Hospital, Ludhiana, Punjab, India; 2 Department of Radiotherapy and Radiation Medicine, Institute of medical Sciences, Banaras Hindu University, Varanasi, India; 3 School of Public Health, J N Medical College, KAHER, Belagavi, Karnataka, India; 4 Centre of Biostatistics, Institute of medical Sciences, Banaras Hindu University, Varanasi, India; 5 Department of Anatomy, Institute of medical Sciences, Varanasi Hindu University, Varanasi, India; 6 Department of Epidemiology and Biostatistics, KLE Academy of Higher Education and Research, Belagavi, Karnataka, India

**Keywords:** cervical cancer, Cox regression, disease-free survival, FIGO stage, India, survival analysis, tumor size

## Abstract

**Introduction:**

Cervical cancer remains a major public health concern in India, with a substantial burden of late-stage diagnosis and limited survival data from tertiary care settings. Disease-free survival (DFS) is a key indicator of treatment effectiveness and recurrence risk, yet evidence on its determinants in Indian populations remains limited. This study aimed to assess DFS pattern and identify prognostic factors among cervical cancer patients treated at a tertiary care center in India.

**Data and methods:**

A retrospective–prospective cohort study was conducted at Sir Sunderlal Hospital, Banaras Hindu University, Varanasi. The study included 587 cervical cancer patients diagnosed between 2011 and 2021, with follow-up until February 2025. Disease-free survival was defined as the time from first follow-up after treatment completion to recurrence. Kaplan–Meier methods, log-rank tests, and Cox proportional hazards models with time-varying covariates were used to estimate survival and identify predictors.

**Results:**

The median follow-up duration was 23.4 months (IQR: 10.2–52.6). Most patients were aged ≤50 years (51.8%), from rural areas (68.5%), and presented with advanced disease (FIGO stage II: 36.3%; stage III: 45.8%). Squamous cell carcinoma accounted for 91.8% of cases, and 76.0% had tumor size ≥4 cm. Kaplan–Meier analysis showed a sharp decline in DFS during early follow-up, indicating a higher risk of recurrence in the initial years. Significant differences in DFS were observed by age (p=0.002), tumor size (p=0.005), vaginal involvement (p=0.011), lymph node involvement (p=0.012), and addiction status (p=0.023). In multivariable analysis, age ≤50 years (AHR = 1.64; 95% CI: 1.16–2.34), tumor size ≥4 cm (AHR = 2.98; 95% CI: 1.44–6.15), and vaginal involvement (AHR = 1.56; 95% CI: 1.06–2.29) were independently associated with poorer DFS. Tumor size also showed a significant time-varying effect (HR = 0.97, p=0.010).

**Conclusion:**

DFS in cervical cancer is strongly influenced by tumor burden and locoregional spread. The early decline in survival highlights the need for intensive follow-up during the first two years post-treatment. Targeted interventions focusing on early detection and risk-stratified management are essential to improve outcomes in resource-limited settings.

## 1 Introduction

Cervical cancer remains a significant global public health concern, particularly in settings, where limited access to HPV vaccination, screening, and timely treatment continues to drive disproportionately high morbidity and mortality. In 2022, an estimated 660,000 new cases and 350,000 deaths were reported worldwide, with nearly 94% of deaths occurring in low- and middle-income countries (LMICs) (1). India contributes substantially to this burden, accounting for approximately 127,000 new cases and over 79,000 deaths annually, with age standardized incidence and mortality rates exceeding global averages (2). Cervical cancer remains a major public health concern in India and its states as well as in districts, with age-standardized incidence and mortality rates of 17.7 and 11.2 per 100,000 women, respectively for India, both exceeding the global averages of 14.1 and 7.1 per 100,000 women (3–5). At the subnational level, data from the Varanasi Population-Based Cancer Registry (2018–2019) indicate a lower but notable burden, with an age-adjusted incidence rate of 9.5 per 100,000 women for cervical cancer (6). Despite being largely preventable, cervical cancer in India is frequently diagnosed at advanced stages, reflecting persistent challenges related to organized screening, public awareness, and timely access to care, with similar barriers observed in other LMICs ( (7).

Disease-Free Survival (DFS) is an important indicator of treatment effectiveness and long-term prognosis (8, 9). It is a robust indicator of treatment efficacy and a key component of long-term prognosis, enabling clinicians and researchers to evaluate the success of interventions such as surgery, radiation, or chemotherapy (9–12) A high DFS rate signifies effective treatment and a reduced risk of recurrence, which is the primary goal of cancer therapy (8, 9). In resource-constrained settings, understanding determinants of DFS is particularly important for optimizing follow-up and improving long-term outcomes.

Although several studies have evaluated survival outcomes in cervical cancer, evidence from Indian populations remains limited and heterogeneous, with variations in follow-up practices and incomplete integration of clinicopathological and socio-behavioral determinants (13–15). This gap impedes the development of risk-stratified surveillance and tailored survivorship interventions. Understanding India’s unique challenges, from high incidence and mortality rates to late-stage diagnoses, is essential for conducting meaningful survival analysis and developing effective strategies to improve long-term outcomes for patients (2).

The present study aimed to characterize disease-free survival trajectories and identify independent determinants of DFS among cervical cancer patients treated at a high-volume tertiary care center in India. We evaluated demographic characteristics, clinical presentation, behavioral factors, reproductive history, tumor characteristics, and treatment-related variables to generate contextually relevant evidence that may support individualized follow-up strategies and inform survivorship planning in resource-limited settings.

## 2 Data and methods

### 2.1 Study area, period and data

A retrospective–prospective cohort study was conducted at Sir Sunderlal Hospital, Banaras Hindu University, Varanasi, Uttar Pradesh, India. Data were obtained from the medical records maintained by the Department of Radiotherapy and Radiation Medicine. The study included patients diagnosed with cervical cancer between 2011 and 2021, with follow-up carried out until February 2025. All eligible consecutive patients during the study period were included. Patients with incomplete clinical records or those diagnosed with cancers other than cervical cancer were excluded. Initially, 648 cervical cancer cases were identified from hospital records. After excluding 33 cases due to incomplete or missing information, and 28 cases excluded due to residual disease, a total of 587 patients were included in the final analysis. Residual disease was defined as persistent clinical and/or radiological evidence of tumor detected within 3 months after completion of primary treatment (16). In this study, recurrence due to cervical cancer was defined as the event of interest. Clinical findings were confirmed by radiological tests to confirm recurrence of disease. The treatment response was evaluated at the first follow-up visit, typically conducted within one month after completion of primary treatment. As clinical and radiological responses to treatment may not be fully evident immediately after treatment completion, this assessment was used to determine remission status. Patients who remained free of recurrence until the end of the follow-up period or were lost to follow-up were treated as censored. Information on recurrence events that occurred before the initiation of data collection in 2022 was obtained from available medical records. Patients who were recurrence-free at the time of data collection continued to be followed prospectively, and information regarding recurrence status and the date of last follow-up was recorded throughout the study period until 2025. Disease-free survival was subsequently measured from the first post-treatment follow-up among patients who achieved complete remission to the date of first documented recurrence or censoring. The outcomes indicated that 140 patients experienced disease recurrence (events), while 447 patients were censored. Patients without relapse at the last follow-up or lost to follow-up were considered censored. Disease free survival was calculated from the date of 1st follow-up of treatment completed to the date of relapse. The median follow-up duration was 23.4 months (IQR: 10.2–52.6 months).

### 2.2 Risk factor

In this study, a range of socio-demographic, clinical, lifestyle, reproductive, pathological, and treatment-related factors were considered as potential risk and prognostic factors for cervical cancer. Socio-demographic characteristics included age at diagnosis, which was grouped into ≤50 years and >50 years. Religion was categorized as Hindu or Muslim, while marital status was classified as married or widowed. Residence was divided into rural and urban areas, and educational status was recorded as illiterate or literate. Clinical presentation at the time of registration was assessed based on common symptoms. These included bleeding per vagina, vaginal discharge, lower abdominal pain, weight loss, and loss of appetite. Each of these variables was recorded as either present or absent. Lifestyle-related factors included dietary habits and addiction status. Diet was categorized as vegetarian or mixed, and addiction was recorded as yes or no, indicating whether the patient had any form of substance use.

Reproductive and obstetric history was also taken into account. Age at menarche was grouped as <14 years and ≥14 years. Age at marriage was categorized as <18 years and ≥18 years. Parity was divided into ≤4 and >4 children. History of abortion was recorded as yes or no. Age at first childbirth was grouped into ≤18 years and >18 years. Mode of delivery was categorized as home delivery or hospital delivery.

Pathological characteristics included histological type, which was classified as squamous cell carcinoma (SCC) or other types (adenosquamous carcinoma, small cell carcinoma, Adenocarcinoma). Tumor size was grouped into <4 cm and ≥4 cm. Parametrium involvement, vaginal wall involvement, and lymph node involvement were each categorized as free, involved, or unknown (information not available). Disease stage was determined using the FIGO staging system and classified into stage I, II, III, IV, and unknown (information not available).

Treatment-related variables included the type of chemotherapy, which was categorized as concurrent chemoradiotherapy (CCRT), neoadjuvant chemotherapy (NACT), or not receiving chemotherapy. Radiotherapy was classified as radical (Radical radiotherapy referred to definitive radiotherapy administered with curative intent as the primary treatment modality, with or without concurrent chemotherapy) or postoperative (Postoperative radiotherapy referred to adjuvant radiotherapy administered following surgical treatment), and brachytherapy was recorded as either received or not received.

### 2.3 Statistical analysis

Survival analysis was performed to evaluate the time-to-event outcome among cervical cancer patients. The primary endpoint of the study was Disease-Free survival, defined as the time (in months) from the date of 1^st^ follow-up after treatment completion to the date of relapse. Patients without relapse at the end of the study period or lost to follow-up were treated as censored observations. The dependent variable was Disease-free survival time, defined as the duration (in months) from the date of 1^st^ follow-up to the date of relapse. The event indicator was coded as 1 = relapse and 0 = censored (not-relapse or lost to follow-up). The independent variables included socio-demographic factors, clinical symptoms, lifestyle factors, reproductive history, pathological variables, and treatment-related variables.

#### 2.3.1 Kaplan–Meier survival analysis

The Kaplan-Meier method was used to estimate the survival probabilities over time. This non-parametric approach allowed for the inclusion of censored observations and provided a graphical representation of survival experience among patients. Survival curves were generated for different categories of explanatory variables such as age group, stage of disease, tumor size, treatment type, and other relevant factors to visualize differences in survival patterns.


$$S(t_{i}\;)=\;\prod_{i:t_{i}\leq t}(1-\;\frac{d_{i}}{n_{i}}\;)$$

Where:

t_i_​ is the time at which the event occurs,

d_i_​ is the number of events (recurrence) at time t_i_​,

n_i_​ is the number of individuals at risk just before time t_i_​.

#### 2.3.2 Log-rank test

To compare survival distributions between different groups, the log-rank test was applied. This test assesses whether there is a statistically significant difference in survival between two or more groups over the entire follow-up period. A p-value of less than 0.05 was considered indicative of a statistically significant difference in survival between groups.


$$\text{χ}2=\sum \;\;\frac{{\left(O_{i}-\;E_{i}\right)}^{2}}{E_{i}}\;\;$$

Where:

O_i_​ is the observed number of events in the group at time t_i_​,

E_i_​ is the expected number of events in the group at time t_i_.

#### 2.3.3 Cox proportional hazards model with time-varying covariates

To identify independent prognostic factors associated with survival, Cox proportional hazards regression analysis was performed. Initially, univariable Cox regression was conducted to assess the association between each explanatory variable and survival outcome. Variables with a p-value less than 0.10 in univariable analysis were considered for inclusion in the multivariable model. The proportional hazards assumption was asses using Schoenfeld residuals. Tumor size violating the proportional hazards assumption were further examined and addressed using time-varying covariates where appropriate. Multicollinearity among variables included in the multivariable Cox regression model was assessed using variance inflation factors (VIF). Categorical variables were converted into dummy variables prior to analysis, and all included predictors demonstrated acceptable collinearity (range from 1.01 to 3.53, with mean VIF of 1.56).

A multivariable Cox proportional hazards with time varying covariates model was then fitted to estimate adjusted hazard ratios (HRs) along with their 95% confidence intervals (CIs), controlling for potential confounders. The hazard ratio represents the relative risk of recurrence associated with each predictor variable.


$$\text{h}(\text{t}|{\text{X}}_{\text{i}})={\text{h}}_{0}(\text{t})\text{ exp }({\text{β}}_{1}{\text{X}}_{1}+{\text{β}}_{2}{\text{X}}_{2}+\cdots +{\text{β}}_{\text{p}}{\text{X}}_{\text{p}})$$

$$\text{h}(\text{t}|{\text{X}}_{\text{i}})={\text{h}}_{0}(\text{t})\text{ exp }[{\text{β}}_{1}*(\text{Age}-\text{group})+{\text{β}}_{2}*(\text{LAP})+{\text{β}}_{3}*(\text{Diet Behavior})+{\text{β}}_{4}*(\text{Addiction})+{\text{β}}_{5}*(\text{Tumor Size})+{\text{β}}_{6}*(\text{Vaginal Involvement})+{\text{β}}_{7}*(\text{LN Involvement})+{\text{β}}_{8}*(\text{Brachytherapy})]$$

where,

- h(t∣X_i_) is the hazard at time t for an individual with covariates X_1_, X_2_,…, X_p_,
- β_1_, β_2,…._, β_7_​ are the regression coefficients estimating the effect of each
- h_0_(t) is the baseline hazard function

## 3 Results

**Table 1** summarizes the baseline characteristics of cervical cancer patients. The majority of women belonged to age ≤ 50 years (51.8%), Hindu (96.4%), married (84.2%), and from rural areas (68.5%), with over half being illiterate (55.4%). Several symptoms were reported at presentation for example more than 8 patient out of 10 (82.1%) reported bleeding per vagina. Furthermore, nearly 79% of the patients reported the vaginal discharge and nearly one fourth reported their appetite loss. A majority followed a mixed diet (64.9%), and 27.1% reported addiction. Early marriage (< 18 years) was common (59.3%), and more than half had parity > 4 (52.1%). Clinically, most patients had squamous cell carcinoma (91.8%) and tumor size ≥ 4 cm (76.0%). A large proportion presented with advanced disease, mainly FIGO stage III (45.8%) and stage II (36.3%). Regarding treatment, neoadjuvant chemotherapy (65.2%) was most commonly used, with most patients receiving radical radiotherapy (70.9%) and brachytherapy (89.1%).

**Table 1:** Baseline clinical and demographic characteristics of the patients.

Background characteristics	Number	Percent
Demographic variable		
Age group		
≤ 50 Years	304	51.8
> 50 Years	283	48.2
Religion		
Hindu	566	96.4
Muslim	21	3.6
Marital status		
Married	494	84.2
Widowed	93	15.8
Residence		
Rural	402	68.5
Urban	185	31.5
Education		
Illiterate	325	55.4
Literate	262	44.6
Complains during registration		
Bleeding P/V		
Yes	482	82.1
No	105	17.9
Discharge P/V		
Yes	461	78.5
No	126	21.5
Lower abdominal Pain		
Yes	265	45.1
No	322	54.9
Weight loss		
Yes	247	42.1
No	340	57.9
Loss of Appetite		
Yes	144	24.5
No	443	75.5
Diet behavior and addiction		
Diet Behavior		
Veg	206	35.1
Mixed	381	64.9
Any type of addiction		
Yes	159	27.1
No	428	72.9
Obstetric history		
Age at Menarche		
< 14	154	26.2
≥ 14	433	73.8
Age at marriage		
< 18	348	59.3
≥ 18	239	40.7
Parity		
≤ 4	281	47.9
> 4	306	52.1
Abortion		
No	248	42.2
Yes	339	57.8
Age at 1st child		
≤ 18	251	44.2
> 18	317	55.8
Total	568	100.0
Mode of Delivery		
Home Delivery	397	69.9
Hospital Delivery	171	30.1
Total	568	100.0
Pathological History		
Histology		
SCC	539	91.8
Other	48	8.2
Tumor Size (cm)		
<4	141	24.0
≥4	446	76.0
Parametrium Involvement		
Free	71	12.1
Involve	431	73.4
Unknown	85	14.5
Walls of vaginal Involvement		
Free	227	38.7
Involve	300	51.1
Unknown	60	10.2
LN involvement		
Free	342	58.3
Involve	188	32.0
Unknown	57	9.7
Figo Stage		
I	37	6.3
II	213	36.3
III	269	45.8
IV	19	3.2
Unknown	49	8.3
Treatment type		
Type of chemotherapy		
CCRT	165	28.1
NACT	383	65.2
No Treatment	39	6.6
Type of Radiotherapy		
Radical	416	70.9
Postop	171	29.1
Brachytherapy		
Yes	523	89.1
No	64	10.9
Total	587	100.0

CM, Centimeter; CCRT, concurrent chemoradiotherapy; NACT, neoadjuvant chemotherapy; SCC, Squamous cell carcinoma.

**Figure 1:**
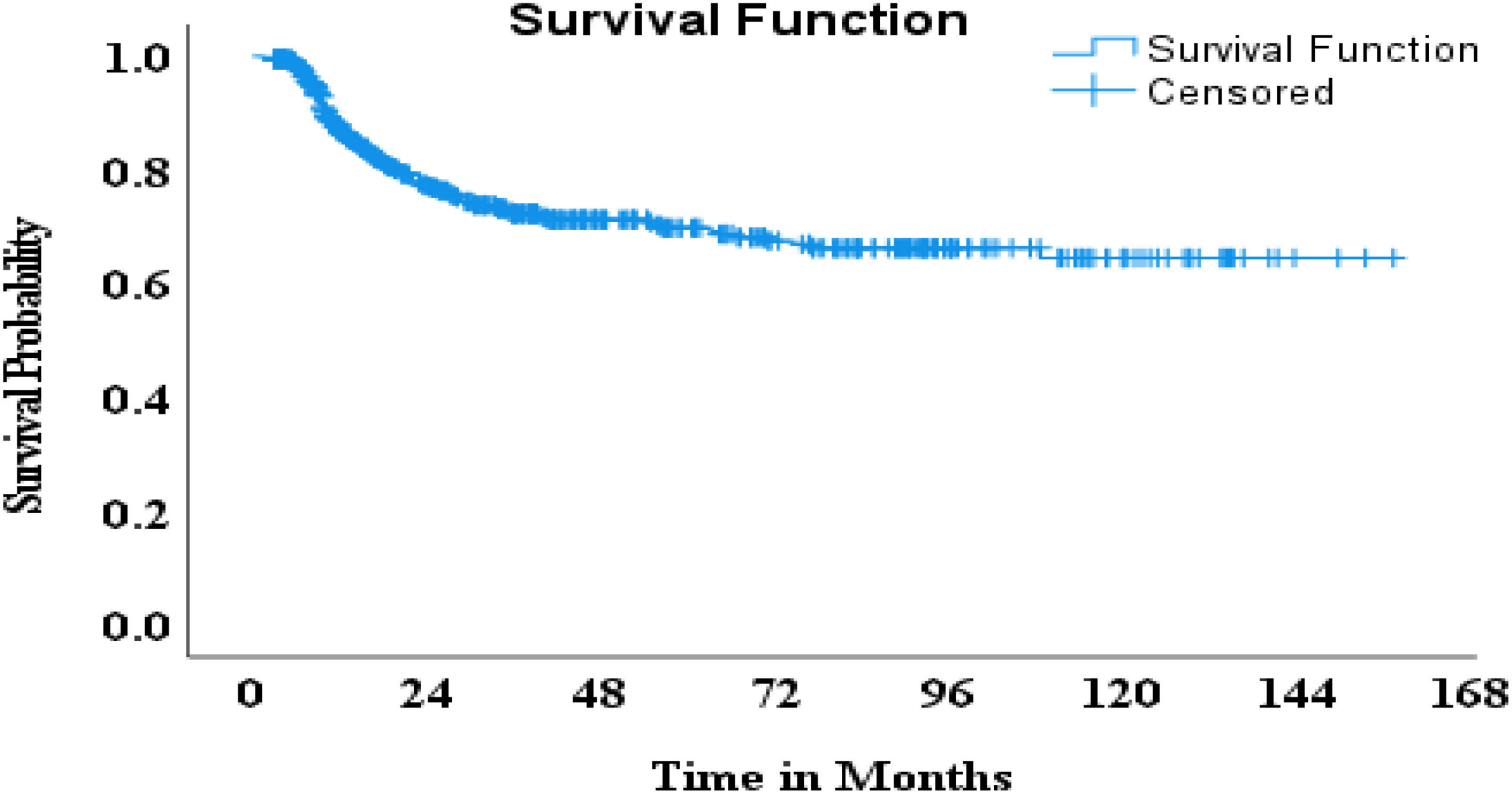
Kaplan–Meier curve of cervical cancer patients for disease-free survival.

Results obtained from Kaplan–Meier curve presented in **Figure 1** shows a progressive decline in disease-free survival over time, with a steeper drop during the early follow-up period, followed by a plateau. The Disease-free survival rates at 1, 3, and 5 years were 86.7%, 72.3%, and 69.8%, respectively. This suggests that recurrences are more frequent in the initial years after treatment.

**Figure 2:**
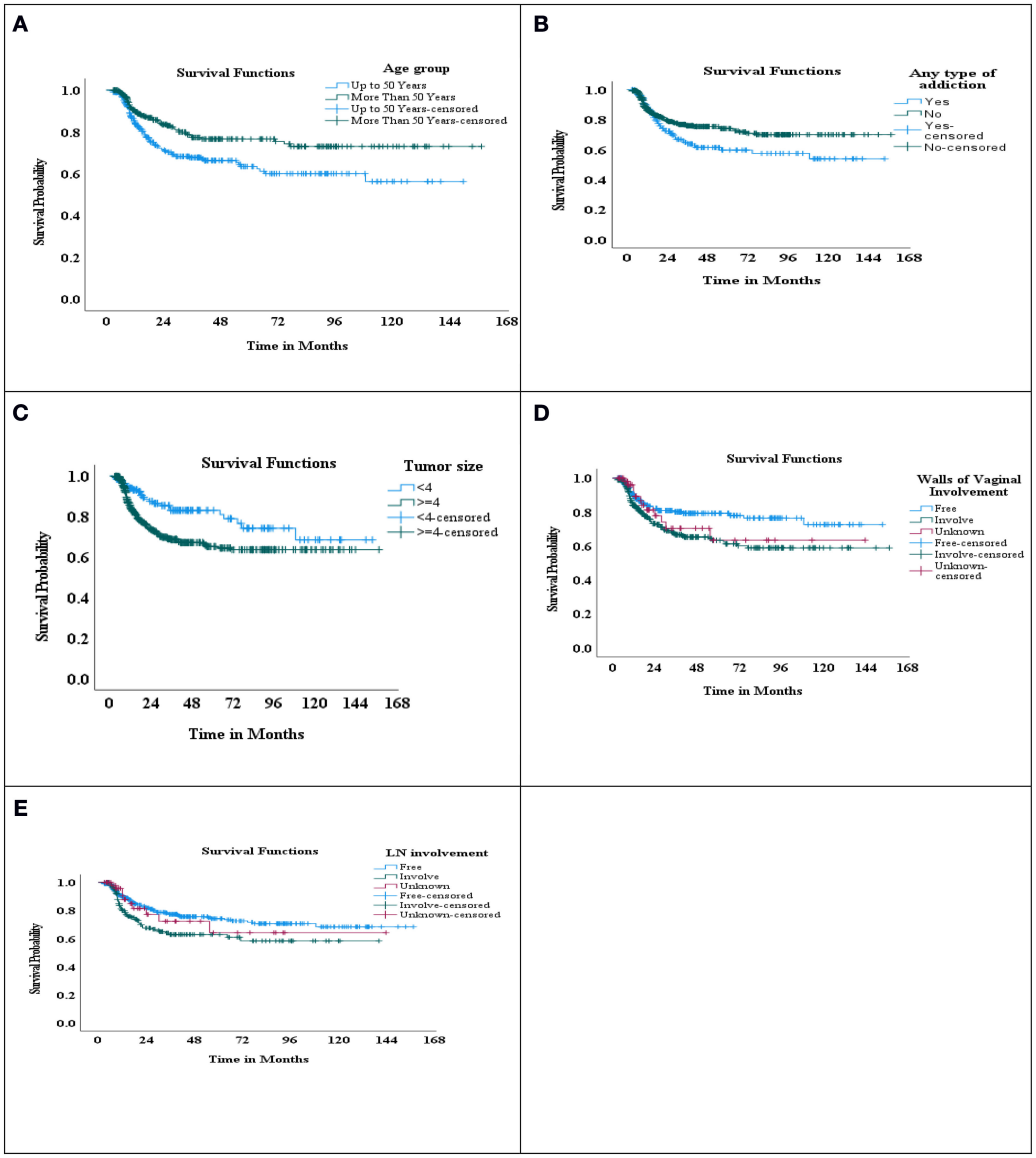
Kaplan–Meier curves showing disease-free survival according to socio-demographic, clinical, behavioral, reproductive, and tumor-related characteristics among cervical cancer patients. **(A)** Kaplan–Meier Curve by Age Group among Cervical Cancer Patients. LR- p-value: 0.002. **(B)** Kaplan–Meier Curve by Addiction Behavior among Cervical Cancer Patients LR-p-value: 0.023. **(C)** Kaplan–Meier Curve by Tumor Size among Cervical Cancer Patients. LR-p-value: 0.005. **(D)** Kaplan–Meier Curve by Vaginal Involvement among Cervical Cancer Patients. LR-p-value: 0.011. **(E)** Kaplan–Meier Curve by Lymph nodes Involvement among Cervical Cancer Patients. LR-p-value: 0.012.

**Figure 2** presents the Kaplan-Meier disease-free survival curves among cervical cancer patients according to various socio-demographic, clinical, behavioral, reproductive, and tumor-related characteristics. For instance, **Figure 2** shows a statistically significant difference in survival across age groups. Patients aged ≤50 years exhibited poorer disease-free survival compared to older patients. The 5-year disease-free survival rate was 63.5% among patients aged ≤50 years, compared with 73.0% among those aged >50 years (p = 0.002). Contrary to general expectations, younger patients showed poorer outcomes, possibly due to more aggressive tumor or delayed diagnosis despite younger age. **Figure 2** finding suggests that any type of addiction is a significant predictor of poorer disease-free survival among cervical cancer patients (5-year survival: 74.0% vs. 59.7%; p = 0.023). Patients with addiction experience a higher risk of recurrence compared to those without addiction.

**Figure 2** shows that the patients with tumor size ≥4 cm had significantly worse disease-free survival. Survival curves presented in **Figure 2** demonstrate a clear separation, where patients with vaginal involvement consistently show reduced disease-free survival compared to those without involvement. The difference is statistically significant (p = 0.011). **Figure 2** The Kaplan–Meier curves show that patients with lymph node involvement have markedly lower survival probabilities compared to those without involvement (5-year survival: 74.2% vs. 63.0%). This difference is statistically significant (p = 0.012). The Kaplan–Meier analyses for the remaining sociodemographic, behavioral, reproductive, clinical, and treatment related factors are presented in **Supplementary Figure 1**.

The unadjusted Cox proportional hazards model (**Table 2**) showed that several factors were significantly related to disease-free survival among cervical cancer patients. Women aged ≤50 years had a higher risk of recurrence compared to older patients (HR = 1.689, p = 0.003). Similarly, patients with any type of addiction were at greater risk (HR = 1.488, p = 0.024). Among the clinical factors, larger tumor size (≥4 cm), vaginal involvement, and lymph node involvement were strongly associated with poorer outcomes, significantly increasing the risk of recurrence. In addition, patients who did not receive brachytherapy had a higher risk of recurrence (HR = 1.803, p = 0.023). On the other hand, most socio-demographic, reproductive, and symptom-related variables did not show a significant association with survival in this unadjusted analysis.

**Table 2:** Univariable and multivariable Cox proportional hazards models with time-varying covariates for disease-free survival in cervical cancer patient.

Background Characteristics	UHR	95% C.I. of HR		P-value	AHR	95% C.I. of HR		P-value
		Lower limit	Upper limit			Lower limit	Upper limit	
Age grp								
>50	Ref.							
<=50	1.689	1.199	2.381	0.003	1.644	1.157	2.336	0.006
Residence								
Urban	Ref.							
Rural	1.299	0.889	1.897	0.177				
Education Status								
Literate	Ref.							
Illiterate	0.861	0.618	1.200	0.378				
Marital Status								
Widow								
Married	1.052	0.648	1.707	0.837				
Religion								
Hindu								
Muslim	1.664	0.814	3.401	0.162				
Bleeding p/v								
No	Ref.							
Yes	0.972	0.631	1.499	0.898				
Discharge p/v								
No	Ref.							
Yes	1.011	0.672	1.522	0.957				
Lower Abdominal Pain								
No	Ref.							
Yes	1.350	0.969	1.881	0.076	1.213	0.867	1.698	0.260
Weight Loss								
No	Ref.							
Yes	1.220	0.874	1.702	0.242				
Loss of Appetite								
No	Ref.							
Yes	1.353	0.929	1.972	0.115				
Diet Behavior								
Veg								
Mixed	1.363	0.947	1.962	0.095	1.390	0.964	2.003	0.078
Any Type of Addiction								
No	Ref.							
Yes	1.488	1.054	2.099	0.024	1.328	0.924	1.908	0.126
Age at menarche								
≥14	Ref.							
<14	1.048	0.720	1.526	0.807				
Age at marriage								
≥18	Ref.							
<18	1.039	0.740	1.459	0.826				
Parity								
≤4	Ref.							
>4	1.098	0.787	1.533	0.582				
Abortion or Miscarriage								
No	Ref.							
Yes	1.066	0.759	1.497	0.711				
Age at birth								
>18	Ref.							
≤18	1.318	0.938	1.852	0.111				
Mode of delivery								
Hospital delivery	Ref.							
Home Delivery	1.124	0.764	1.654	0.553				
Histology								
SCC								
Others	1.439	0.855	2.422	0.171				
Tumor Size (cm)								
<4	Ref.							
≥4	1.850	1.191	2.871	0.006	2.976	1.440	6.149	0.003
Parametrium Involvement								
Free								
Involve	1.634	0.900	2.966	0.107				
Unknown	1.835	0.890	3.786	0.100				
Vaginal Involvement								
Free								
Involve	1.755	1.210	2.545	0.003	1.557	1.060	2.289	0.024
Unknown	1.372	0.720	2.612	0.336	1.439	0.509	4.066	0.493
LN Involvement								
Free								
Involve	1.678	1.186	2.374	0.003	1.417	0.983	2.041	0.062
Unknown	1.140	0.587	2.211	0.699	0.934	0.310	2.812	0.903
Figo Stage								
I	Ref.							
II	1.645	0.654	4.136	0.290				
III	2.425	0.980	6.003	0.055				
IV	2.878	0.832	9.959	0.095				
Unknown	1.971	0.660	5.888	0.224				
Chemotherapy								
CCRT	Ref.							
NACT	1.207	0.825	1.767	0.333				
Radiotherapy								
Radical	Ref.							
Postop	1.172	0.815	1.683	0.392				
Brachytherapy								
Received	Ref.							
Not Received	1.803	1.085	2.997	0.023	1.311	0.769	2.235	0.320
TVC								
				Tumor Size	0.970	0.948	0.993	0.010

CM, Centimeter; CCRT, concurrent chemoradiotherapy; NACT, neoadjuvant chemotherapy; SCC, Squamous cell carcinoma.

After adjusting for other factors using a Cox model with time-varying covariates (**Table 2**.2), only a few variables remained important predictors of survival. Age ≤50 years continued to be predicted with a higher risk (AHR = 1.644, p = 0.006). Tumor size ≥4 cm emerged as the strongest predictor, with nearly three times higher risk of recurrence (AHR = 2.976, p = 0.003). Vaginal involvement also remained significantly associated with poorer survival (AHR = 1.557, p = 0.024). Although lymph node involvement showed some effect, it was not statistically significant after adjustment (p = 0.062). Importantly, tumor size showed a significant time-varying effect (HR = 0.970, p = 0.010), indicating that its changing impact of survival over the follow-up period. Variables such as any type of addiction, diet, and treatment factors no longer significance after adjustment, suggesting that their effects were influenced by other clinical variables. Overall, the results highlight that tumor burden and disease spread are the key factors affecting survival, and considering time-varying effects gives a more realistic understanding of how these factors influence outcomes over time.

## 4 Discussion

In this retro-prospective cohort of 587 cervical cancer patients treated at Sir Sunderlal Tertiary Care Hospital, with a median follow-up of 23.4 months (IQR: 10.2-52.6), we observed a distinct pattern of disease-free survival (DFS), characterized by an early decline followed by a plateau over time. Survival analysis demonstrated that younger age (≤50 years), tumor size ≥4 cm, and vaginal involvement emerged as important correlates of reduced disease-free survival. Patients with parametrial involvement and advanced FIGO stage demonstrated a trend toward poorer disease-free survival; however these did not reach statistical significance. While lymph node involvement and addiction showed associations in unadjusted analyses, these effects attenuated after multivariable adjustment. Interestingly, tumor size also demonstrated a significant time-varying effect, suggesting that its influence on recurrence risk may change over the course of follow-up. This variation may also be attributed to the variation in the tumor size over time. This finding highlights the dynamic nature of prognostic factors in cervical cancer and may have implications for the timing and intensity of post-treatment surveillance.

The observed early decline in DFS suggests that recurrence risk is highest during the initial post-treatment period, consistent with evidence indicating that the majority of recurrences occur within the first few years after treatment, with nearly half occurring within two years (17). This pattern underscores the importance of intensive follow-up during this critical period.

The relationship between age and DFS has been inconsistently reported, with some studies reporting no significant relationship (18, 19). In contrast, younger age (≤50 years) was independently associated with reduced disease-free survival in the present cohort. This unexpected finding should be interpreted with caution. Although the observed association persisted after adjustment for measured covariates, it may reflect residual confounding, treatment heterogeneity, differences in disease presentation, or unmeasured clinical characteristics rather than a direct biological effect of younger age itself. Further studies incorporating more detailed tumor and treatment characteristics are needed to better understand this association.

Behavioral factors such as addiction showed an association with DFS in unadjusted analysis but did not remain significant after adjustment. Previous studies have reported that alcohol consumption and smoking are associated with poorer survival outcomes in cervical cancer (20–22); however, these effects may be mediated through disease stage, treatment, and other clinical factors. This suggests that addiction may act as a distal determinant rather than an independent prognostic factor.

Not all variables associated with cervical cancer risk or prognosis necessarily exert a direct influence on disease-free survival. Behavioral and reproductive factors may influence outcomes indirectly through disease occurrence, healthcare access, treatment pathways, or overall health status rather than recurrence per se. The lack of statistical significance for variables such as addiction and obstetric history in the present study may therefore reflect their role as distal determinants rather than independent predictors of DFS. Treatment-related variables, including chemotherapy regimen, radiotherapy approach, and brachytherapy, did not retain statistical significance after multivariable adjustment. This attenuation may reflect confounding by indication, whereby treatment decisions are inherently influenced by disease stage, tumor size, and other clinicopathological characteristics that are themselves strong determinants of recurrence. These findings should therefore not be interpreted as evidence of a lack of treatment effect but rather as highlighting the complexity of disentangling treatment effects from baseline disease severity in observational analyses.

Tumor-related factors emerged as key determinants of DFS. Patients with tumor size ≥4 cm experienced substantially lower disease-free survival, which is consistent with prior evidence linking larger tumor size to increased tumor burden and reduced treatment response (23–26). However, one study reported no independent association between tumor size and DFS (18), suggesting that its prognostic role may be influenced by coexisting factors such as stage and nodal involvement.

Vaginal and parametrial involvement represent locoregional spread beyond the cervix and may pose challenges in achieving optimal local control (18, 27). In the present study, vaginal involvement retained independent prognostic significance after adjustment. Previous evidence suggests that vaginal extension is associated with an increased risk of distant metastasis, including lymph node involvement, even in the absence of parametrial invasion (28).

Although patients with parametrial involvement in our cohort demonstrated a tendency toward reduced disease-free survival, the association did not reach statistical significance. Nevertheless, previous studies have identified parametrial invasion as an adverse prognostic factor associated with recurrence and poorer survival outcomes (18, 27). The dense lymphatic and vascular networks within the parametrium may facilitate tumor dissemination (29), providing a biologically plausible explanation for these observations.

The findings should also be interpreted in the context of the FIGO staging system, which remains the cornerstone of prognostication in cervical cancer (23) (Han et al., 2024). Although disease-free survival declined numerically with advancing FIGO stage, the association did not remain statistically significant after adjustment. This attenuation may reflect overlap with correlated clinicopathological characteristics rather than the absence of a clinically meaningful relationship. Lymph node involvement, a marker of regional disease spread, is a well-established prognostic factor in cervical cancer (23, 24, 30). Increasing nodal burden, particularly ≥3 metastatic lymph nodes, has consistently been linked to inferior disease-free and overall survival (23, 24). However, lymph node involvement did not retain statistical significance after adjustment in the present study. This may reflect its interrelationship with other markers of disease severity included in the model. Additionally, lymph node status was categorized as free, involved or unknown, without accounting for nodal burden or location, which may have limited the ability to detect its independent prognostic effect.

The findings of this study have important clinical implications. Tumor size ≥4 cm and vaginal involvement may help identify patients at higher risk of recurrence, who could benefit from risk-stratified and more intensive follow-up, particularly during the early post-treatment period. These results also underscore the importance of early detection and timely treatment to prevent disease progression and improve outcomes in cervical cancer.

An important consideration when interpreting the present findings is the variability in disease-free survival definitions across the literature. While some studies define DFS as the time to recurrence or death, others define it based solely on recurrence, metastatic progression, or treatment failure (17, 19, 22). The present study defined DFS based on documented recurrence following treatment completion. Consequently, direct comparisons of survival estimates across studies should be interpreted with caution. Nevertheless, the prognostic importance of tumor-related factors identified in the present study is broadly consistent with previous reports evaluating disease-free or recurrence-free survival in cervical cancer.

The study has certain limitations. Being conducted at a single tertiary care center, the findings may have limited generalizability to other populations. Additionally, the retrospective component of the study introduces the possibility of residual confounding and information bias, as some relevant clinical or treatment-related variables may not have been fully captured. Missing information for certain clinicopathological variables, including lymph node and parametrial involvement, was retained as separate “unknown” categories to preserve the study sample. Although, the multicollinearity diagnostics did not indicate substantial collinearity among the included variables, the possibility of residual interrelationships between covariates and their potential influence on the estimated associations cannot be entirely excluded. While this approach minimized data loss, it may have reduced the precision of some estimates and influenced the observed associations. Future studies with more complete data capture and appropriate approaches for handling missing data may help further strengthen the robustness of these findings. Finally, the relatively modest sample size may have limited the statistical power to detect independent associations for some variables, including parametrial involvement.

## 5 Conclusion

In conclusion, disease-free survival in cervical cancer is influenced by a combination of tumor-related and clinical factors, with larger tumor size and vaginal involvement emerging as important prognostic indicators in this population. The observed steep early decline in DFS, with a substantial proportion of recurrences likely within the first two years, highlight a critical window for recurrence. These findings underscore the need for vigilant, risk-stratified follow-up during the initial post-treatment period while reinforcing the importance of early diagnosis and timely treatment to improve patient outcomes.

## Data Availability

The data underlying the findings presented in this study are not publicly available due to ethical and institutional restrictions. The data contain sensitive patient information, and access is limited to authorized researchers within the institution for confidentiality and compliance purposes. Requests for access to the data should be directed to the corresponding author or the Institutional Ethics Committee of Banaras Hindu University. Requests to access the datasets should be directed to bajarang7894@gmail.com.
