# Supplementary material for: Survival beyond treatment: patterns and determinants of disease-free survival in cervical cancer at a tertiary care center in India
Source: Front Oncol. 2026 Jul 10;16:1872738. doi: 10.3389/fonc.2026.1872738 (PMC13395714; doi:10.3389/fonc.2026.1872738)
Supplement: Supplementary file 1 [file Table1.docx]

**Survival Beyond Treatment: Patterns and Determinants of Disease-Free Survival in Cervical Cancer at a Tertiary Care Center in India**

***Supplementary Materials***

**Table S1.** Kaplan-Meier Estimates of 1-, 3-, and 5-Years Disease-free Survival Probabilities among Cervical Cancer Patients

| **Variable** | **Survival Rate** | | | **Number of Event** | **p-value** |
| --- | --- | --- | --- | --- | --- |
|  | **1 Year** | **3 Years** | **5 Years** |  |  |
| **Overall Survival** | 86.7 | 72.3 | 69.8 | 140 |  |
| **Age group** | | | | | |
| <=50 | 84.1 | 67.7 | 63.5 | 88 | 0.002 |
| >50 | 89.6 | 77.3 | 73 | 52 |  |
| **Residence** |  |  |  |  |  |
| Urban | 89 | 76.2 | 73.6 | 36 | 0.175 |
| Rural | 86.3 | 70.7 | 68.8 | 104 |  |
| **Education Status** | | | | | |
| Literate | 85.5 | 69.6 | 68.5 | 67 | 0.377 |
| Illiterate | 88.5 | 74.6 | 70.8 | 67 |  |
| **Marital Status** | | | | | |
| Widow | 84.3 | 71.4 | 71.4 | 121 | 0.837 |
| Married | 87.6 | 72.5 | 69.6 | 19 |  |
| **Religion** | | | | | |
| Hindu | 86.8 | 73.3 | 70.7 | 132 | 0.158 |
| Muslim | 95 | 47.5 | 47.5 | 8 |  |
| **Bleeding p/v** | | | | | |
| Yes | 87.1 | 73.1 | 69.9 | 115 | 0.898 |
| No | 87.3 | 68.8 | 68.8 | 25 |  |
| **Discharge p/v** | | | | | |
| Yes | 87.6 | 72.1 | 70.4 | 111 | 0.957 |
| No | 88.2 | 73.2 | 67.7 | 29 |  |
| **Lower Abdominal Pain** | | | | | |
| Yes | 84.8 | 69.9 | 66.1 | 72 | 0.074 |
| No | 89.1 | 74.3 | 72.8 | 68 |  |
| **Weight Loss** | | | | | |
| Yes | 85.5 | 70.9 | 66.6 | 63 | 0.241 |
| No | 88.7 | 73.4 | 72 | 77 |  |
| **Loss of Appetite** | | | | | |
| Yes | 83.3 | 64.6 | 62.6 | 37 | 0.114 |
| No | 87.7 | 74.5 | 71.8 | 103 |  |
| **Diet Behavior** | | | | | |
| Veg | 88.9 | 78.2 | 76.2 | 41 | 0.094 |
| Mixed | 86.2 | 69.2 | 66.3 | 99 |  |
| **Any Type of Addiction** | | | | | |
| Yes | 88.3 | 63.8 | 59.7 | 51 | 0.023 |
| No | 87.6 | 75.9 | 74 | 89 |  |
| **Age at menarche** | | | | | |
| ≥14 | 87.6 | 72.2 | 69 | 103 | 0.807 |
| <14 | 86 | 72.6 | 70.4 | 37 |  |
| **Age at marriage** | | | | | |
| >=18 | 85.4 | 71.7 | 70.5 | 55 | 0.825 |
| <18 | 88.7 | 72.8 | 69.5 | 85 |  |
| **Parity** | | | | | |
| ≤4 | 87 | 73.9 | 72 | 62 | 0.581 |
| >4 | 87.3 | 70.9 | 67.9 | 78 |  |
| **Abortion or Miscarriage** | | | | | |
| Yes | 87 | 71 | 68.8 | 85 | 0.711 |
| No | 87.4 | 74.3 | 71.2 | 55 |  |
| **Age at birth** | | | | | |
| >18 | 88.8 | 76.4 | 74.1 | 66 | 0.11 |
| ≤18 | 85.3 | 68.9 | 66 | 67 |  |
| **Mode of delivery** | | | | | |
| Hospital delivery | 84.1 | 76.3 | 72.2 | 35 | 0.552 |
| Home Delivery | 88.7 | 71.9 | 68.3 | 98 |  |
| **Histology** | | | | | |
| SCC | 87.2 | 73 | 70 | 124 | 0.169 |
| Others | 86.2 | 65.5 | 61.6 | 16 |  |
| **Tumor Size** | | | | | |
| <4 | 94 | 83.1 | 83.1 | 24 | 0.005 |
| ≥4 | 84.8 | 68.6 | 65.2 | 116 |  |
| **Parametrium Involvement** | | | | | |
| Free | 90.6 | 82.6 | 82.6 | 12 | 0.211 |
| Involve | 87.5 | 71.3 | 68.7 | 109 |  |
| Unknown | 86.8 | 70.8 | 61.7 | 19 |  |
| **Vaginal Involvement** | | | | | |
| Free | 90.2 | 80.1 | 79 | 41 | 0.011 |
| Involve | 84 | 66.6 | 63.3 | 87 |  |
| Unknown | 96 | 70.4 | 63.4 | 12 |  |
| **LN Involvement** | | | | | |
| Free | 90.3 | 77.3 | 74.2 | 72 | 0.012 |
| Involve | 80.5 | 63 | 63 | 58 |  |
| Unknown | 90.5 | 72.3 | 64.5 | 10 |  |
| **Figo Stage** | | | | | |
| I | 88.1 | 84.7 |  | 5 | 0.102 |
| II | 90.9 | 77.1 | 74.1 | 47 |  |
| III | 84.4 | 67 | 64 | 74 |  |
| IV | 71.8 | 62.8 | 62.8 | 5 |  |
| Unknown | 88.8 | 71.8 | 62.8 | 9 |  |

**Figures S1:** Kaplan–Meier Survival Curves for Disease-Free Survival According to Sociodemographic, Clinical, and Treatment-Related Factors among Cervical Cancer Patients

| Figure S1.1. Kaplan–Meier Curve by Religion among Cervical Cancer Patients  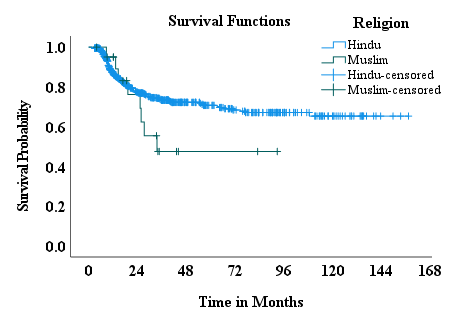  LR-p-value: 0.158 | Figure S1.2. Kaplan–Meier Curve by Marital Status among Cervical Cancer Patients  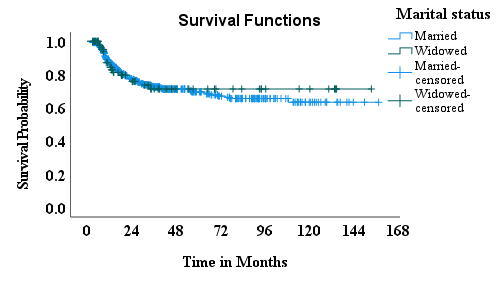  LR-p-value: 0.837 |
| --- | --- |
| Figure S1.3. Kaplan–Meier Curve by Residence among Cervical Cancer Patients  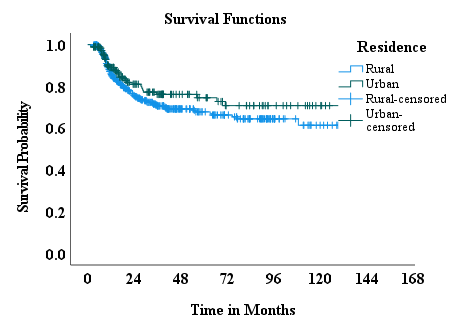  LR-p-value: 0.175 | Figure S1.4. Kaplan–Meier Curve by Educational Status among Cervical Cancer Patients  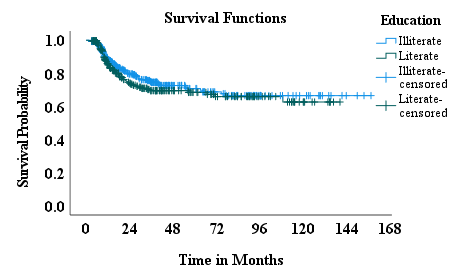  LR-p-value: 0.377 |
| Figure S1.5. Kaplan–Meier Curve by Bleeding p/v among Cervical Cancer Patients  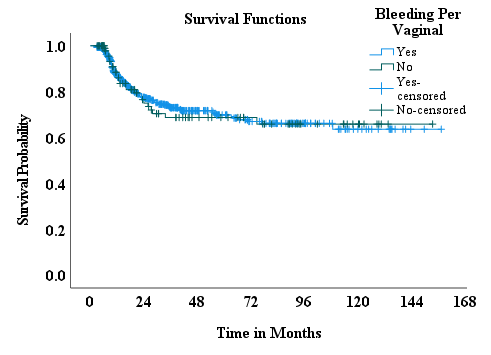  LR-p-value: 0.898 | Figure S1.6. Kaplan–Meier Curve by Discharge p/v among Cervical Cancer Patients  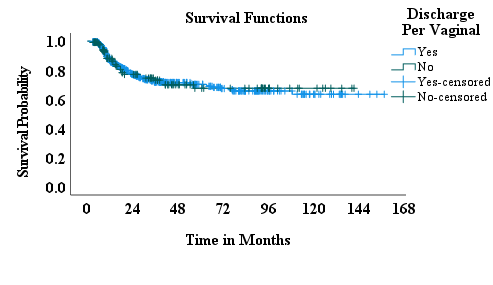  LR-p-value: 0.957 |
| Figure S1.7. Kaplan–Meier Curve by Lower abdominal pain among Cervical Cancer Patients  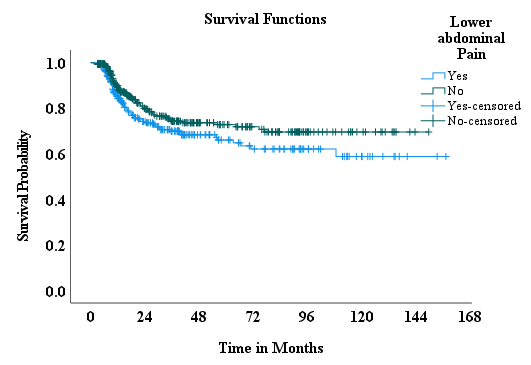  LR-p-value: 0.074 | Figure S1.8. Kaplan–Meier Curve by Weight loss among Cervical Cancer Patients  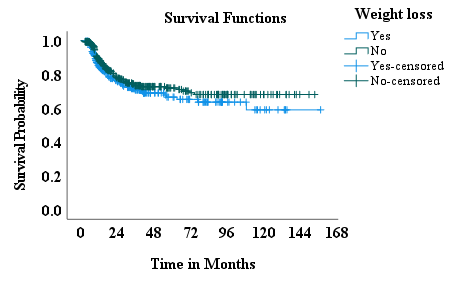  LR-p-value: 0.241 |
| Figure S1.9. Kaplan–Meier Curve by Loss of appetite among Cervical Cancer Patients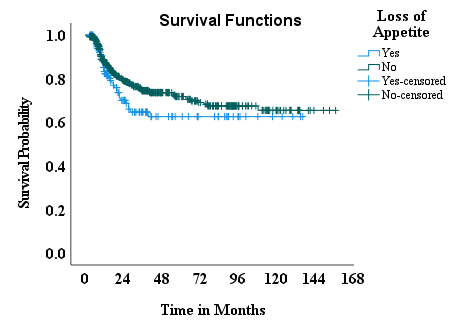  LR-p-value: 0.114 | Figure S1.10. Kaplan–Meier Curve by Diet behavior among Cervical Cancer Patients  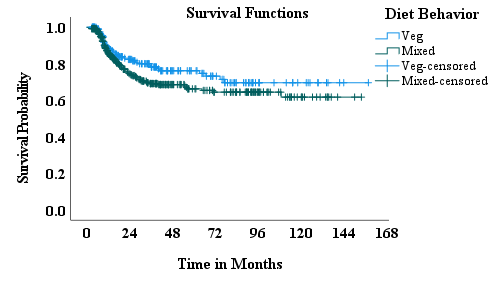  LR-p-value: 0.094 |
| Figure S1.11. Kaplan–Meier Curve by Age at menarche among Cervical Cancer Patients  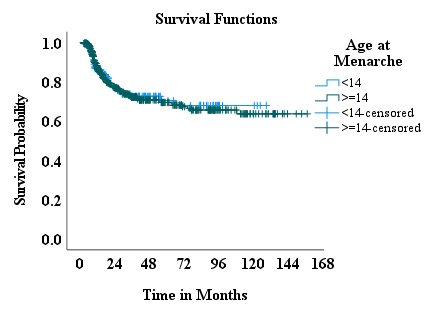  LR-p-value: 0.807 | Figure S1.12. Kaplan–Meier Curve by Age at marriage among Cervical Cancer Patients  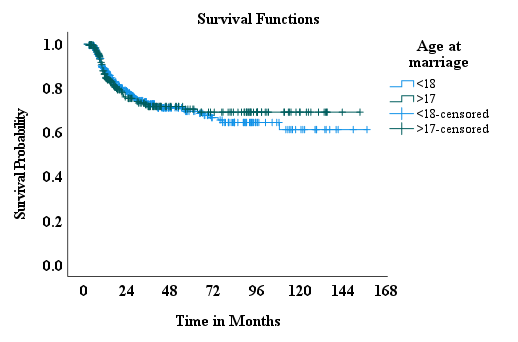  LR-p-value: 0.825 |
| Figure S1.13. Kaplan–Meier Curve by Parity among Cervical Cancer Patients  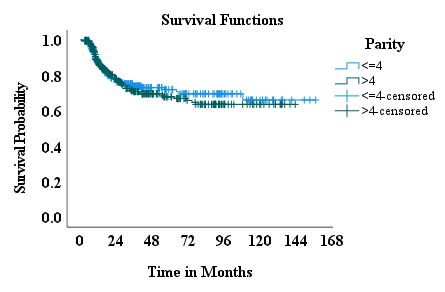  LR-p-value: 0.581 | Figure S1.14. Kaplan–Meier Curve by history of Abortion or miscarriage among Cervical Cancer Patients  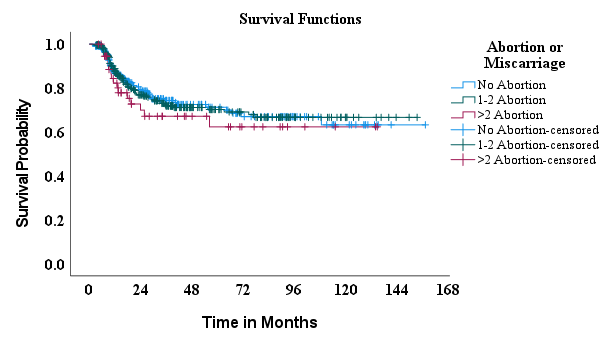  LR-p-value: 0.626 |

| Figure S1.15. Kaplan–Meier Curve by Age at 1^st^ Birth among Cervical Cancer Patients  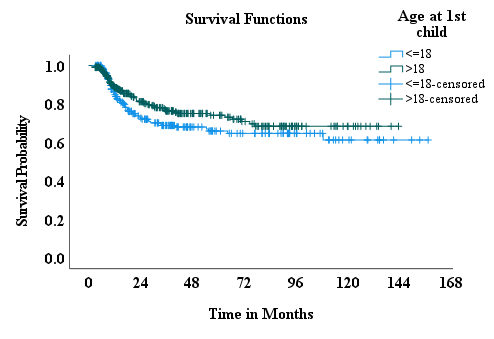  LR-p-value: 0.110 | Figure S1.16. Kaplan–Meier Curve by Mode of Delivery among Cervical Cancer Patients  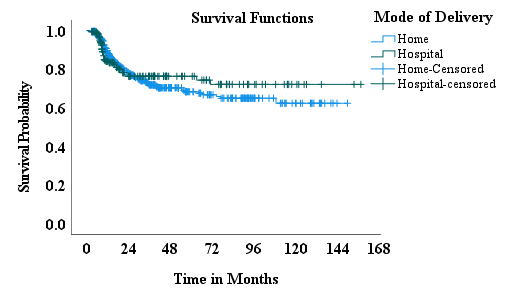  LR-p-value: 0.552 |
| --- | --- |
| Figure S1.17. Kaplan–Meier Curve by Parametrium Involvement among Cervical Cancer Patients  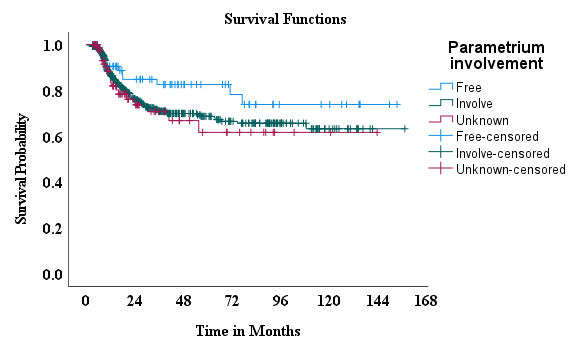  LR-p-value: 0.211 | Figure S1.18. Kaplan–Meier Curve by FIGO Stage among Cervical Cancer Patients  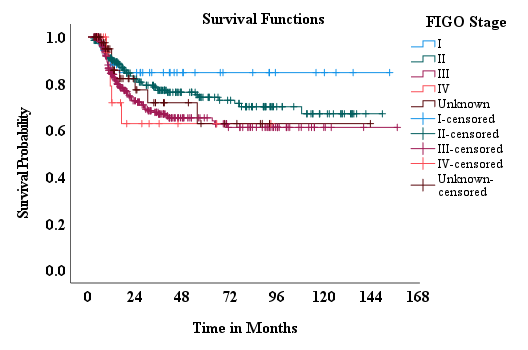  LR-p-value: 0.102 |
